# Effects of COVID-19 lockdown on weight in a cohort of allergic children and adolescents

**DOI:** 10.1186/s13052-022-01273-y

**Published:** 2022-06-10

**Authors:** Giulia Brindisi, Vincenza Patrizia Di Marino, Francesca Olivero, Daniela De Canditiis, Giovanna De Castro, Anna Maria Zicari, Caterina Anania

**Affiliations:** 1 Department of Mother-Child, Urological Science, Sapienza University of Rome, 00185 Rome, Italy; 2 Pediatric Clinic, Department of Pediatrics, Fondazione IRCSS Policlinico San Matteo, University of Pavia, 27100 Pavia, Italy; 3 Institute of Applied Calculus-CNR Rome, 00185 Rome, Italy

**Keywords:** COVID-19 pandemic, Weight gain, Lockdown, Consolatory-food, Pediatric age

## Abstract

**Background:**

COVID-19 lockdown caused sudden changes in people’s lifestyle, as a consequence of the forced lockdown imposed by governments all over the world. We aimed to evaluate the impact of lockdown on body mass index (BMI) in a cohort of allergic children and adolescents.

**Methods:**

From the first of June until the end of October 2020, we submitted a written questionnaire to all the patients who, after lockdown, carried out a visit at the Pediatric Allergy Unit of the Department of Mother-Child, Urological Science, Sapienza University of Rome. The questionnaire was composed by 10 questions, referring to the changes in their daily activities. Data were extrapolated from the questionnaire and then analyzed considering six variables: BMI before and BMI after lockdown, sugar intake, sport, screens, sleep, and anxiety.

**Results:**

One hundred fifty-three patients agreed to answer our questionnaire. Results showed a statistically significant increase in the BMI after lockdown (20.97 kg/m2 ± 2.63) with respect to the BMI before lockdown (19.18 kg/m2 ± 2.70). A multivariate regression analysis showed that the two variables that mostly influenced the increase in BMI were sleep and anxiety.

**Conclusions:**

For the analyzed cohort of allergic children and adolescents we obtained significant gain in BMI as consequences of lockdown, which can be explained by many factors: high consumption of consolatory food, less sport activities, more time spent in front of screens, sleep alteration associated with increased anxiety. All these factors acted together, although sleep alteration and increased anxiety were the most influential factors that led to the worsening or the onset of weight gain, creating the basis for future health problems.

**Supplementary Information:**

The online version contains supplementary material available at 10.1186/s13052-022-01273-y.

## Introduction

By March 2020, the World Health Organization (WHO) declared a global pandemic of coronavirus disease 2019 (COVID-19), caused by severe acute respiratory syndrome coronavirus 2 (SARS-CoV-2), that emerged in Wuhan (China) in December 2019 and then rapidly spread all over the world [1] .

SARS-CoV-2 reveals marked tropism for the respiratory tract often leading to airway diseases with dyspnea, dry cough, sore throat, rhinitis and in few cases to respiratory failure. The acute respiratory distress (ARDS) is responsible for admission to intensive care, especially for patients with comorbidities, who have a higher mortality rate [2–5]. Other possible SARS-CoV-2 symptoms are fever, anosmia, ageusia, fatigue, gastrointestinal symptoms and skin disorders [2–5].

However, pediatric patients often present mild symptoms, and only a small percentage develop severe/critical illness [6].

Neverthless, the appearance of SARS-CoV-2 variants, that seem to affect children and adolescents most intensely, does not have to be overlooked [7]. Given the high rate of SARS-CoV-2 contagiousness, the worrying viral infectivity lead governments and health authorities in several countries to put in place restrictions to reduce viral spread, influencing people’s lifestyles for the first time ever.

Italy was quarantined from the beginning of March until the end of May 2020, when a national lockdown was put in place to flatten the curve of contagions. This strict lockdown was associated with drastic changes in lifestyle habits for the entire population, with limitations of individual freedom and the onset of psychological issues [8], causing in addition to health and social discomfort, an economic crisis worldwide [9].

Among the containment measures, commercial activities, restaurants and sports centers were closed, as well as schools, setting up distance learning for the first time [10].

People were only allowed to go out for serious and urgent health reasons; thus, several patients affected by chronic disease, like allergies, were followed up by phone or emails.

Among chronic diseases, allergies are not to be considered as risk factors for developing SARS-CoV-2 infection as long as they are controlled; hence the need to continue standard treatment during the pandemic, according to the underlying allergic pathology [11–13].

Nevertheless one of the most common consequences of this tight lockdown was weight gain and the possible worsening of a pre-existing obesity among children, adolescents and adults [14, 15].

In the pediatric age, the evaluation of weight adequacy is carried out through the use of body mass index (BMI) centile curves; value higher than the 85th centile is an indication of overweight, value higher than the 97th centile it is an indication of obesity [16, 17]. In particular, the WHO growth reference curves defines BMI-for-age Z-scores, defining overweight as > 1 but ≤2 standard deviation (SD) of the WHO growth reference median, and obesity as > 2 SD of the WHO growth reference median [18].

To the best of our knowledge, no studies have reported data on weight gain in a cohort of allergic children and adolescents during COVID-19 lockdown until now, considering the changes in their lifestyle.

We aimed to evaluate the impact of SARS-CoV-2 home quarantine on BMI among our cohort of allergic patients followed at the Pediatric Allergy Unit of the Department of Mother-Child, Urological Science, Sapienza University of Rome, by submitting a written questionnaire (lifestyle questionnaire) with a particular focus on changes in their habits during the lockdown.

## Methods

From the first of June until the end of October 2020, we proposed a written questionnaire to children and adolescents (aged between 6 and 16 years) who, after SARS-COV-2 lockdown, carried out a follow-up visit at the Pediatric Allergy Unit of the Department of Mother-Child, Urological Science, Sapienza University of Rome .

After each visit made for a specific allergic pathology (allergic rhinitis (AR), asthma, urticaria, anaphylaxis, dermatitis, conjunctivitis and/or food allergy) we gained parents’ verbal consent for the submission of a “lifestyle questionnaire” to their children.

The questionnaire was made up of 10 questions, structured in different sections (see supplementary material). The first part consisted of personal information (name/surname, the allergic pathology for which they visited our clinic.

We used a mechanical weight assessment scale (SECA 700) equipped with an altimeter for the assessment of the height on the occasion of the two visits both before and after the lockdown. Only the month of the visit was recorded and not its exact date. Of the 153 patients, 62 made the visit in June (15 days on average after the end of the lockdown), 32 in July, 14 in August, 36 in September and 9 in October, with this information we can calculate an average of about 55 days after the end of the lockdown. The second part of the lifestyle questionnaire reported some questions with a positive or negative answer (yes or not) regarding: eating habits during lockdown, sports activities (made or not at home), daily time spent in front of screens (less or more than 8 hours for each day), sleep behavior (less or more than 8 hours per night), feelings of anxiety (present or not). The patient had to fill in the questionnaire with the aid of a parent. No financial incentive was offered.

### Statistical analysis

The collected data were extrapolated from the questionnaire and then analyzed using the software R, which is a free software environment for statistical computing, available at https://www.r-project.org/. For each subject we have six measurements/variables: BMI before, BMI after, sugar intake, sport activity, use of screens, sleep, and anxiety. The first two being continuous variables, the last five being Bernoulli variables. Firstly, we conducted a descriptive analysis of the last five variables, reporting for each of them the number of children who answered yes and the number of children who answered no. Secondly, we applied the Shapiro-Wilk test to the first two variables to check for gaussianity, and hence we applied a paired t-test to the BMI before and after lockdown. Finally, we applied a multivariate linear regression model where the response Y is the difference in BMI before and after lockdown and the covariates are sugar intake, sports, screens, sleep, and anxiety.

## Results

A total of 153 patients have agreed to answer our questionnaire; all subjects were aged between 6 and 16 with mean 9.6 and standard deviation 3.3. The population consisted of 74 female children (48%) and 79 male children (52%). None of the enrolled patients had COVID-19 during lockdown.

The pathologies affecting the children involved are reported in Table 1 below, represented in percentages.

**Table 1:** Description of the pathologies of the enrolled patients

Pathology	Number of children	Percentage of number of children
Asthma	17	11.11%
Rhinitis	60	39.22%
Dermatitis	13	8.5%
Urticaria	3	1.96%
Food allergy and rhinitis	4	2.61%
Asthma and rhinitis	42	27.45%
Rhino-conjunctivitis	14	9.15%

Description of the pathologies by which the enrolled patients are affected expressed in percentages

**Fig. 1:**
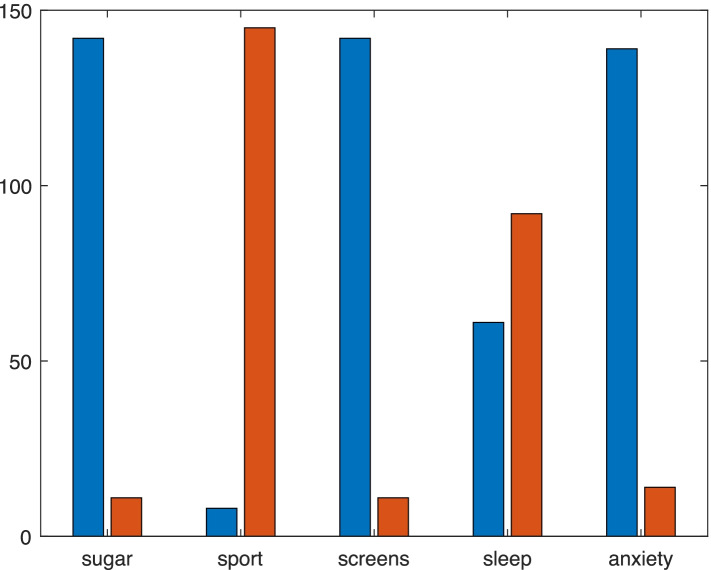
Bar plot of all the variables considered in the lifestyle questionnaire. Bar plots of the five variables considered in the lifestyle questionnaire: -Number of subjects who did not increase the amount of sugar intake (red = 0), number of subjects who increased the amount of sugar intake (blu = 1); − of subjects who did not present feeling of anxiety (red = 0), number of subjects who presented feeling of anxiety (blu = 1); −Number of subjects who slept less than 8 hours per night (red = 0), number of subjects who slept 8 hours per night or more (blu = 1); −Number of subjects who did not practice sport at home (red = 0), number of subjects who made sport at home (blu = 1); −Number of subjects who spent more than 8 hours per day in font of screens (red = 0), number of subjects who spent less than 8 hours per day in front of screens (blu = 1)

As a first step, we conducted a descriptive analysis of five variables considered in the lifestyle questionnaire. We summarized results in Fig. 1, where we reported the number of children in the y-axis and for each variable, two columns.

The red columns represent the number of children that did not change the amount of sugar consumption, the hours of sleep, the hours spent practicing sports, the hours spent looking at screens and those who did not experience anxiety or fear, while the blu columns represent those who changed these habits and who experienced anxiety and fear. In particular, among our population, we observed an increase in the consumption of sweets equal to 92.81% (142 children); only 7.19% (11 children) did not change their dietary intake of sweets. Considering indoor activities, 94.77% (145 children) did not play any kind of sports, instead only 5.23% (8 children) practiced sports at their home. At the same time 92.81% (142 children) increased the time spent in front of screens (such as PCs, tablets or TV) more than 8 hours per day and only 7.19% (11 children) did not change the amount of time per day spent in front of them. Moreover, considering an average of 8 hours sleep per night a percentage equal to 60.13% (92 children) reduced the number of hours of sleep per night, while the 39.87% (61 children) slept the same number of hours during the lockdown period. The feeling of “fear or anxiety” affected 90.85% of the patients (139 children), while a percentage equal to 9.15% (14 children) did not report these feelings.

As a second step of our analysis, we applied the Shapiro-Wilk test to the first two variables, obtaining *p* = 0.18 and *p* = 0.47 respectively for the BMI before and BMI after, hence we accepted the null hypothesis of Gaussianity for both variables with any reasonable significant level. The mean of the BMI after lockdown was 20.97 kg/m2 ± 2.63. The mean of the BMI before lockdown was 19.18 kg/m2 ± 2.70. Since the two standard deviations are almost equals, we applied a two-tailed paired t-test obtaining a *p*-value << 0.001.

With these values, the effect size of the paired t-test is 0.67, which with sample size 153 gives a power of almost 1.

**Fig. 2:**
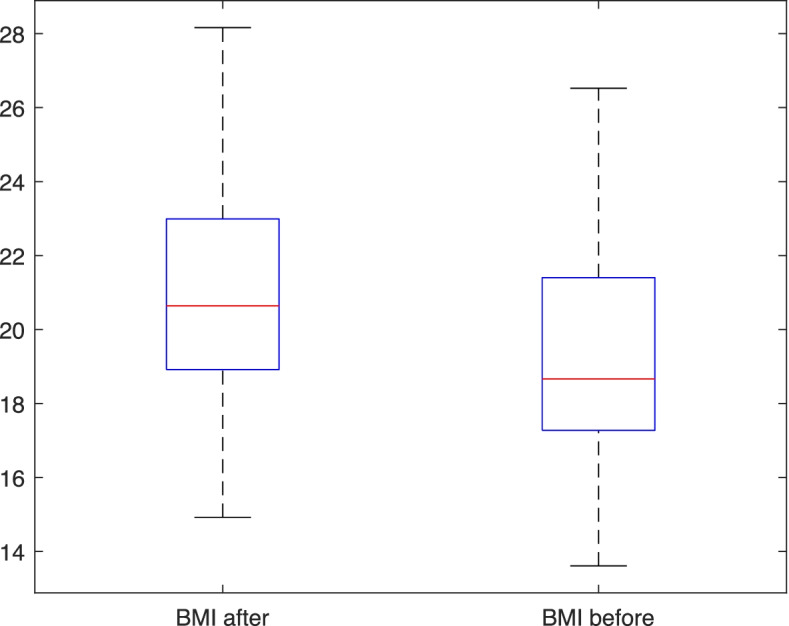
Boxplot of the variables BMI after and BMI before the lockdown. In each box, the central mark indicates the median, and the bottom and top edges of the box indicate the 25th and 75th percentiles, respectively. The whiskers extend to the most extreme data points not considered outliers

We report the boxplot of the BMI after and before the lockdown in Fig. 2, we can conclude that there was a statistical significant increment of the BMI during the lockdown period.

As a final step, we performed a multivariate linear regression analysis, to study the influence of the five lifestyle variables on the BMI’s variation. In other words, we applied a multivariate linear regression model with response variable Y=BMI_after-BMI_before, and covariates sugar, sports, screens, sleep, and anxiety. We report in Table 2 the obtained estimated coefficients with their *p*-values, with an overall *p*-value of the F-statistic vs. constant model of 0.12.

**Table 2:** Estimated regression coefficients with *p*-values

	Estimate	***p***-value
**Intercept**	1.2532	4.6486e-05
sugar	0.19777	0.30993
sport	0.05785	0.80579
screens	0.14221	0.49367
**sleep**	**−0.2318**	**0.033678**
**anxiety**	**0.33895**	**0.058189**

Multivariate linear regression model with response variable Y=BMI_after-BMI_before, and covariates sugar, sport, screens, sleep, and anxiety

Setting the significance level at 0.1, then we can conclude that not all lifestyle variables significantly influence the increment of BMI when jointly considered, and only the last two variables (sleep and anxiety) have significant *p* values. To validate this observation, we performed a bivariate linear regression analysis, with covariates sleep and anxiety, obtaining for the sleep variable a regression coefficient equal to − 0.206 (*p*-value = 0.045), for the anxiety variable a coefficient equal to 0.361 (*p*-value = 0.038) and an overall *p*-value of the F-statistic vs. constant model of 0.025. Hence, we can conclude that, from a statistical point of view, the increase in hours of sleep negatively influenced the increase of BMI, while the increased anxiety influenced positively the increase of BMI.

## Discussion

COVID-19 outbreak has significantly altered the daily lifestyle and routine of children and adolescents, with critical implications for their quality of life with consequences for their physical and mental health [19].

The recent systematic review and meta-analysis conducted by Tu-Hsuan Chang et al., after analyzing 12 studies, concluded about the significant increases in body weight and BMI among school-age children and adolescents during COVID-19 lockdown [15] .

Overweight and obesity, common consequences of lockdown, have been considered as an independent risk factor for severe COVID-19; this has been related to endothelial dysfunction associated with metabolic syndrome, and the increased expression of angiotensin-converting enzyme 2 (ACE2) receptors, the entrance door for SARS-COV-2 into the cells [20].

Results from our COVID-19 lifestyle questionnaire have shown a statistically significant increase in the BMI after lockdown compared to the BMI before it due to the sudden changes in daily life habits with repercussions both on mental and physical state. Different cofactors may have contributed, acting together in different ways [21, 22].

Home quarantine, among our patients, have led to an increased consumption of “consolatory food”, a higher daily time spent in front of screens for both school and leisure activities and a reduction in the time spent doing sport activities at home. In addition, anxiety was the result of hearing bad news from the media, regarding the spread of the virus and the related deaths it has caused [21–23] .

Polly Wait et al., conducted an online survey among a cohort of school-aged children and adolescents of 4 and 16 years of age, evaluating their mental health during UK lockdown(March and May 202). They concluded that the findings were concerning in terms of impact on children and youth’s mental health for whom it was therefore critical to provide effective support [24].

Anxiety generated also an altered sleep quality which led to an increased perception of hunger resulting in more consumption of sweets and caloric food as “consolatory function” [14, 21, 22].

“Craving for food” is related to the satisfaction in carbohydrates consumption that releases serotonin with a positive effect on mood [14].

It is known that stress influences mood and lifestyle, but also stimulates the sense of hunger especially for sweet foods. In fact, two behaviors have been described during stressful situations: increased food intake or food refusal [25].

Thus, a vicious circle was triggered: the increase in feeling of anxiety had a negative effect on sleep with a consequent increase in the sensation of hunger; as well as the inability to engage in physical activity and more time spent in front of screens led to further stress and weight gain with possible consequences, such as cardiovascular and metabolic complications [26, 27].

Thus the prolonged lockdown negatively impacted children’s physical and mental health [28]; in fact, it is enough to consider, among all the cited factors above, the impossibility to practice physical exercise, which has evident beneficial effects on body weight and mental health [29].

In addition, as reported by Pappa et al. in their systematic review, a high percentage of behavioral and sleep disturbances, as a consequence of sleep deprivation, increase cortisol levels leading to insulin resistance and an increase in visceral fat [30].

Also Liu Y et al., in support of this fact, in their online survey during COVID-19 lockdown, reported that the interruption of daily activities, sports and socialization had a crucial role in generating anxiety and fear among children and adolescents, associated with the news about contagions and deaths given by mass media [31].

The systematic review conducted by Jones EAK et al., reported instead the negative effect that the pandemic is having especially on adolescent mental health, emphasizing the need to therapies and resources capable of supporting adolescents for their physical and mental growth [32].

In addition, Oliver Ramos-Álvarez et al., have conducted a survey on a cohort of 50 Spanish children of 11/12 years of age, and they found a significant differences in the anthropometric parameters, the BMI and body fat percentage of pre- and post-lockdown (*p* < 0.05) [33].

According to the literature, the results of our study evaluated body weight and BMI before and after COVID-19 lockdown in a cohort of allergic patients, analyzing, through a lifestyle questionnaire, the changes in their daily activities.

We found a general trend of reduced physical activity, as well as increased time spent in front of screens, altered sleep quality, higher consumption of consolatory food, and the onset of anxiety feelings. Multivariate analysis showed that not all these covariates considered were equally able to influence BMI change; subsequent bivariate linear regression showed that sleep and anxiety were the two covariates responsible for statistically significant differences, albeit with an inverse value of this relation. In detail, increased sleep time negatively influenced the BMI increase, whereas increased anxiety positively influenced this increase.

This study presents some limitations. In fact there are some recall bias (systematic errors) due to the fact that the answers to the questionnaires were given on the basis of memories and/or sensations experienced by the children months before. However, such recall bias were limited by the fact that the parent supervised the child’s responses.

## Conclusions

Weight gain is one of the most common consequences of COVID-19 lockdown among children and adolescents, and this study confirm this tendency for our allergic patients. This seems to stem from many factors, which acted together, creating a vicious circle that led to the worsening or to the onset of weight gain with development of health problems that may continue into adulthood and are to be avoided in childhood.

## Supplementary Information

**Additional file 1.** COVID-19 lifestyle questionnaire.

## Data Availability

The datasets used and/or analyzed during the current study are available from the corresponding author on reasonable request.

## Abbreviations

BMI: Body mass index
COVID-19: Coronavirus disease 2019
SARS-CoV-2: Severe acute respiratory syndrome coronavirus 2
ACE2: Angiotensin-converting enzyme 2
