# Supplementary material for: Effects of COVID-19 lockdown on weight in a cohort of allergic children and adolescents
Source: Ital J Pediatr. 2022 Jun 10;48:88. doi: 10.1186/s13052-022-01273-y (PMC9187144; doi:10.1186/s13052-022-01273-y)
Supplement: Supplementary file 1 — Additional file 1. COVID-19 lifestyle questionnaire. [file 13052_2022_1273_MOESM1_ESM.docx]

**Questionnaire:**

**Name:……..**

**Surname:…….**

**Date of birth…..**

**1. Disease:**

| **Pathology** |
| --- |
| Asthma |
| Rhinitis |
| Dermatitis |
| Urticaria |
| Food allergy and rhinitis |
| Asthma and rhinitis |
| Rhino-conjunctivitis |

**2. Body weight (kg)  before the lockdown:…………**

**3. Height (cm)  before the lockdown:………..**

**4. Body weight (kg) after the lockdown: ………**

**5. Height (cm) after the lockdown: ………..**

**6. Diet: Did you increase your consumption of sweets or consolatory food (pizza, pasta…) etc… during the lockdown?**

- **Yes**
- **Not**

**7. FEELING OF ANXIETY: Did you experience feelings of anxiety during the lockdown?**

- **Yes**
- **Not**

**8. SLEEPING TIME:** **How much total time did you sleep per night during the lockdown?**

- **> 8 hours a day**
- **<8 hours a day**

**9. SPORT: Did you play any sports at home during the lockdown?**

- **Yes**
- **Not**

**10. SCREEN TIME: How much total time did you spend in front of screens (TV, PC, tablet) during the lockdown?**

- **> 8h per day**
- **< 8h per day**
